# Survival outcomes of surgery in patients with pulmonary large-cell neuroendocrine carcinoma: a retrospective single-institution analysis and literature review

**DOI:** 10.1186/s13023-021-01730-7

**Published:** 2021-02-12

**Authors:** Yeye Chen, Jiaqi Zhang, Cheng Huang, Zhenhuan Tian, Xiaoyun Zhou, Chao Guo, Hongsheng Liu, Shanqing Li

**Affiliations:** Department of Thoracic Surgery, Peking Union Medical College Hospital, Chinese Academy of Medical Sciences & Peking Union Medical College, Beijing, 100730 China

**Keywords:** Pulmonary large-cell neuroendocrine carcinoma, Surgical treatment, Prognosis

## Abstract

**Background:**

Pulmonary large-cell neuroendocrine carcinoma (pLCNEC) is a very rare malignancy originating from the lung and bronchus, and its biological behaviour, clinical diagnosis, treatment and prognosis are poorly understood. Thus, the clinical characteristics and surgical treatment-related prognostic factors of this rare disorder must be explored.

**Results:**

The clinical data of 59 patients (48 males and 11 females) who were treated by surgery and diagnosed with pLCNEC by postoperative pathology at Peking Union Medical College Hospital from April 2004 to April 2019 were analysed retrospectively. The median patient age was 62 years (38–79 years), and the median duration of disease was 2 months (0.5–18 months). Compared with other lung malignancies, pLCNEC lacks specific clinical symptoms and imaging features, and preoperative biopsy pathology is often insufficient to confirm the diagnosis. The corresponding numbers of patients who were classified into stages I, II, III and IV according to the postoperative pathological tumour-nodal-metastasis stage were 25, 12, 15 and 7, respectively. The median overall survival was 36 months (0.9–61.1 months). The 1-year, 3-year and 5-year survival rates were 76.3%, 49% and 44.7%, respectively. The tumour stage exerted a significant effect on survival (Cox multivariate analysis p < 0.05).

**Conclusions:**

For patients with resectable pLCNEC, multidisciplinary therapy based on surgery may have good survival benefits, and tumour stage is an independent risk factor for the prognosis of pLCNEC.

## Background

Pulmonary large-cell neuroendocrine carcinoma (pLCNEC) is a very rare malignant tumour originating from the lung and bronchus. It was first reported in 1989 and summarized by Travis et al. [1]. In 2015, pathological classification by the World Health Organization (WHO) regarded LCNEC as one of four subtypes of bronchopulmonary neuroendocrine carcinoma: typical carcinoid, atypical carcinoid, small-cell lung cancer (SCLC) and LCNEC [1–3]. pLCNEC is an aggressive malignancy with a poor prognosis and accounts for 2–3% of all lung cancers [4, 5]. Due to the short time this pathology has been known and the fact that it is a rare disease, little is known about its biological behaviour, clinical diagnosis, or treatment. Here, we retrospectively analysed the clinical data of 59 patients with pLCNEC confirmed by postoperative pathology at Peking Union Medical College Hospital (PUMCH) in the last 15 years. Combining our data with the findings of other previously reported cohort studies on the surgical treatment of pLCNEC, we summarized the curative effect of comprehensive treatment based on surgery and analysed the potential prognostic factors.

## Results

### General characteristics

From April 2004 to April 2019, 59 patients were diagnosed with pLCNEC by postoperative pathology at PUMCH, including 48 males and 11 females. The median age was 62 years (38–79 years), and the median duration of disease (from the time of onset to the time of operation) was 2 months (0.5–18 months). Forty-three patients (72.9%) had a history of smoking. Four patients (6.8%) had a family history of malignancy (Table 1).

**Table 1:** Clinical characteristics of 59 pLCNEC patients

Variables	Value (N = 59)
Sex ratio (male:female)	48:11
Median age (years)	62 (38–79)
Median disease duration (months)	2 (0.5–18)
Smoking/non-smoking	43:16
Family history of malignancy	4 (6.8%)
Initial symptoms	
Asymptomatic	25 (42.4%)
Cough	16 (27.1%)
Bloody sputum	12 (20.3%)
Chest and back pain	4 (6.8%)
Fever	3 (5.1%)
Chest tightness	4 (6.8%)
Median tumour size (cm)	3.0 (1.0–12.0)
Peripheral type/central type	36:23
Scope of surgical resection	
Lobectomy	41 (69.5%)
Combined lobectomy	13 (22.0%)
Sublobar resection	5 (8.5%)
Surgical approach	
VATS	23 (39.0%)
PLT	36 (61.0%)
Pathological type (pure:mixed)	51:8
Postoperative TNM stage	
I	25 (42.4%)
II	12 (20.3%)
III	15 (25.4%)
IV	7 (11.9%)
Median Ki-67 index	72.5 (40–95)
AT	
nd	15 (25.4%)
Ct	35 (59.3%)
Rt	1 (1.7%)
Ct and Rt	8 (13.6%)
Perioperative complications	
Pulmonary infection	2 (3.4%)
Respiratory failure	1 (1.7%)
Acute cerebral infarction	2 (3.4%)
Air leakage	1 (1.7%)
Arrhythmia	1 (1.7%)

*VATS* video-assisted thoracic surgery, *PLT* posterolateral thoracotomy, *nd* not done, *AT* adjuvant therapy, *Ct* chemotherapy, *Rt* radiotherapy

The scope of surgical resection indicates the extent of lung parenchyma resection.

### Clinical symptoms and tumour parameters

Compared with those of patients with other types of lung cancer, the clinical manifestations of patients in this group lacked specificity. The common symptoms included cough in 16 cases (27.1%), bloody sputum in 12 cases (20.3%), chest and back pain in 4 cases (6.8%), chest tightness in 4 cases (6.8%) and fever in 3 cases (5.1%), while 25 cases showed no symptoms (42.4%) (Table 1).

Our cohort did not show any specific chest computed tomography (CT) features that were meaningful for the differential diagnosis of other types of lung cancer. There were 36 (61.0%) peripheral cases and 23 (39.0%) central cases shown by CT imaging. The median tumour size was 3.0 cm (1.0–12.0 cm). Among all the enrolled patients, 20 patients underwent preoperative bronchoscopy or CT guided biopsy, and only 3 cases (15%) were considered LCNEC. Other diagnoses included 4 cases of undifferentiated carcinoma (20%), 4 cases of poorly differentiated adenocarcinoma (20%), 3 cases of SCLC (15%), one case of squamous-cell carcinoma (5%), one case of adenosquamous carcinoma (5%), one case of combined SCLC (5%) and 3 cases of necrosis and inflammation (15%). In addition, 5 patients underwent preoperative chemotherapy.

Increased tumour markers, including CYFRA211, SCCAg, NSE, ProGRP and CEA, were detected before surgery in 18.8% (6/32), 0.0% (0/26), 34.4% (11/32), 33.3% (8/24) and 39.3% (11/28) of our patients, respectively.

### Surgery and pathology

The main surgical procedures included lobectomy (41 cases), combined lobectomy (lobectomy + lobectomy or sublobar resection) (13 cases) and sublobar resection (wedge resection or segmental resection) (5 cases). Thoracoscopic surgery and posterolateral thoracotomy were performed on 23 and 36 patients, respectively.

Thirty-one patients underwent an intraoperative rapid frozen pathology examination, via which only 1 (3.2%) patient was confirmed to have LCNEC, 1 (3.2%) patient was diagnosed with carcinoid cancer, 4 patients (12.9%) were diagnosed with neuroendocrine tumours (unclassified), 14 (45.2%) patients were diagnosed with non-small-cell lung cancer (NSCLC) and 11 (35.5%) patients were diagnosed with poorly differentiated carcinoma. According to postoperative pathology, 51 patients were diagnosed with pure pLCNEC, and 8 patients were diagnosed with combined pLCNEC, including 4 patients with combined adenocarcinoma, 3 patients with combined squamous-cell carcinoma and one patient with combined atypical carcinoid.

No patients died during the perioperative period. 7 patients developed complications during the perioperative period, including 2 cases of pulmonary infection, 2 cases of acute cerebral infarction, one case of respiratory failure, one case of air leakage and one case of arrhythmia (Table 1). All the patients mentioned above recovered well and were discharged after active treatment.

According to postoperative pathological tumour-nodal-metastasis (TNM) staging system, the numbers of patients who were classified into stage I, II, III and IV were 25 (42.4%), 12 (20.3%), 15 (25.4%) and 7 (11.9%), respectively.

The Ki-67 index of 34/59 patients was assessed by postoperative pathology. The median value was 72.5% (40–95%). Immunohistochemical indicators were also collected retrospectively. The positive rates of AE1/AE3, cluster of differentiation 56(CD56)/natural killer 1, synaptophysin, chromogranin A, thyroid transcription factor-1 and p63 were 88.2% (30/34), 81.1% (30/37), 94.4% (51/54), 73.2% (41/56), 76.2% (32/42) and 3.6% (1/28), respectively.

### Postoperative treatment, follow-up and prognosis

**Fig. 1:**
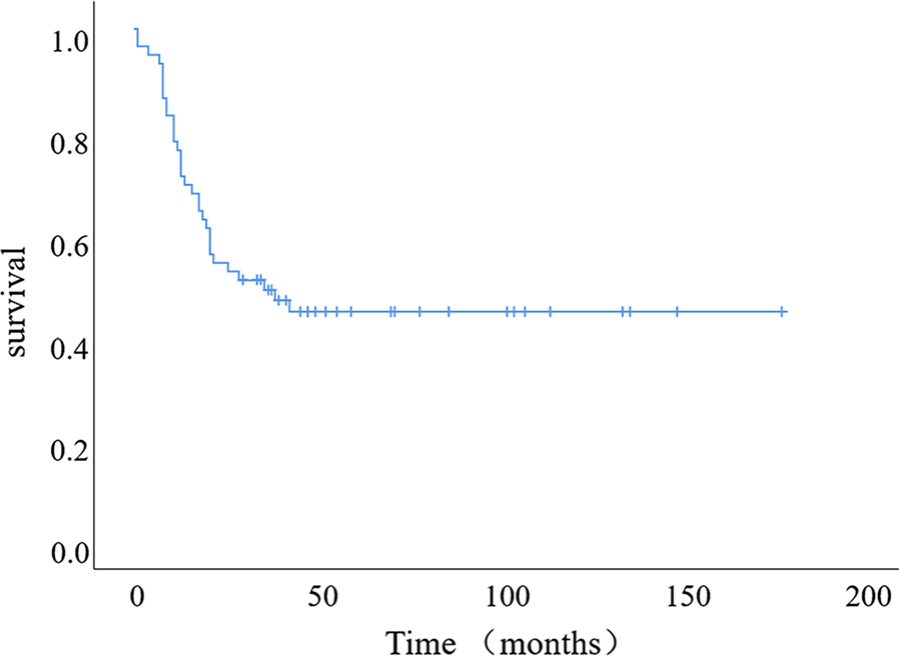
Survival analysis by the Kaplan–Meier method and the survival curve of the whole group. The median OS was 36 months (10.9–61.1 months), and the 1-year, 3-year and 5-year OS rates were 76.3%, 49.0% and 44.7%, respectively

Fifteen patients did not receive postoperative adjuvant therapy, including 10 stage I patients, 3 stage II patients, one stage III patient and one stage IV patient. By the end of follow-up, 7 out of 15 patients died, including 2 stage I patients, 3 stage II patients, one stage III patient, and one stage IV patient. The remaining 44 patients received postoperative adjuvant treatment with platinum-based chemotherapy regimens, of which 26 died and 18 survived. Metastatic recurrence was the commonest pattern of tumour progression, and the sites of recurrence were mainly bone, brain and lymph nodes (mainly on the supraclavicular sites and mediastinum). The Kaplan–Meier method was used to calculate overall survival (OS), and the median survival time was 36 months (10.9–61.1 months). The 1-year, 3-year and 5-year OS rates were 76.3%, 49.0% and 44.7%, respectively (Fig. 1). Univariate log-rank analysis suggested that the surgical approach, tumour size (dichotomized around the median), TNM stage and preoperative adjuvant therapy were factors affecting OS. Other factors, such as age, smoking history, family history, lesion location, elevated tumour markers and postoperative adjuvant therapy, showed no statistically significant differences. For stage I patients, adjuvant therapy had no significant effect on survival (p = 0.054). The surgical approach, tumour size (dichotomized around the median), N stage, M stage and neoadjuvant therapy were included in the Cox multivariate model, the results of which suggested that the surgical approach (HR 0.407, 95% CI 0.195–0.851, p = 0.017), N stage (HR 1.689, 95% CI 1.042–2.740, p = 0.034) and M stage (HR 6.712, 95% CI 2.229–20.211, p = 0.001) were independent risk factors for OS in pLCNEC patients (Table 2).

**Table 2:** Univariate log-rank analysis and Cox multivariate analysis of prognostic factors

Risk factor	Univariate log-rank analysis (*p*)	Multivariate analysis	
		*p*	HR (95.0% CI)
Age	0.563		
Sex (median as cut-off)	0.180		
Smoking history	0.326		
Duration (median as cut-off)	0.297		
Surgical approach (PLT versus VATS)	*0.004*	*0.017*	0.407 (0.195–0.851)
Surgical scope (sublobar resection versus lobectomy and combined lobectomy)	0.933		
Tumour location (peripheral type versus central type)	0.942		
Pathological type (pure versus mixed)	0.471		
T stage	*0.000*		
N stage	*0.002*	*0.034*	1.689 (1.042–2.740)
M stage	*0.000*	*0.001*	6.712 (2.229–20.211)
Tumour size (median as cut-off)	*0.004*	*0.278*	1.637 (0.672–3.983)
TNM stage	*0.000*		
Complication (yes versus no)	0.411		
AT (all)	0.586		
AT (stage I)	0.054		
Neoadjuvant therapy	*0.037*	0.416	0.605 (0.181–2.028)

*PLT* posterolateral thoracotomy, *VATS* video-assisted thoracic surgery, *HR* hazard ratio, *CI* confidence interval, *AT* adjuvant therapy, p < 0.05 indicated that there was significant difference

### Literature review

Reports on surgical treatments of pLCNEC have been retrieved in PubMed since 2004, and the survival and prognostic factors of pLCNEC patients reported by the various studies were listed in Table 3. There was a significant increase in the number of associated studies in the last five years. pLCNEC patients were mainly elderly male smokers. The smoking rate in one study was < 50% but in most studies it was 80–90%, and the median age was over 60 years in almost all studies. The median survival time varied from 24.1 months to 54.4 months, and the 5-year survival rate was 29–58%. The main prognostic factors were pathological stage, T stage or tumour size, N stage, adjuvant chemotherapy and age. Other occasionally reported factors included tumour distribution (peripheral or central), pneumonectomy, plasma albumin concentration, NSE concentration, C-kit protein expression, Nestin expression and *EGFR* mutation.

**Table 3:** Literature review of the survival of pLCNEC patients

Author	Year	Cases	Age	Sex (M/F)	Smoking rate (%)	Country/region (institutions)	5-year survival	Median OS	Stage	Potential prognostic factors
Casali et al. [6]	2004	33	63.5	31/2	95	Italy (SI)	51%	Na	I–IIIA	C-kit protein expression
Doddoli et al. [7]	2004	20	62	18/2	85.0	France (SI)	36%	49	ALL	None (due to small number of patients)
Veronesi et al. [8]	2006	144	63	117/27	94	Italy (MI)	42.5%	Na	I–III	Age, stage III, pneumonectomy
Ryuge et al. [9]	2012	30	67	27/3	93.3	Japan (SI)	47%	28.4	I–III	Nestin expression
Fournel et al. [10]	2013	63	64	49/14	88.8	France (SI)	49.2%	Na	ALL	pT stage
Zhang et al. [11]	2015	50	59	47/3	70	China (SI)	58%	49.3 (DFS)	I–III	Serum Alb and NSE levels
Eichhorn et al. [5]	2015	57	62.4	41/16	94.7	Germany (SI)	50% (3-yr)	36	ALL	pN stage, expression of CD56 and chromogranin-A
Roesel et al. [12]	2016	127	63.8	82/45	97.6	Germany (SI)	53.8%	Na	All	pT and pN stages
Kim et al. [13]	2017	139	64	126/13	83.5	Korea (SI)	53%	Na	all	Adjuvant treatment (for stage II or higher)
Okui et al. [14]	2017	26	68.8	23/3	96.2	Japan (SI)	Stage I: 86.7% stage > I: 45.5%	54.4	I–IIIA	NLR, pathological stages
Filosso et al. [15]	2017	400	66	252/148	24.8	ESTS (MI)	45%	43	all	Age, performance status, TNM stage, adjuvant chemotherapy
Eichhorn et al. [16]	2018	76	60.9	54/22	97.0	Germany (SI)	29%	Na	all	Pathological N stage, PD-L1 expression
Zhou et al. [17]	2018	126	64	118/8	57.1	China (SI)	50.4% (3-year)	44.5	All	Tumour location, *EGFR* mutation
Ohtaki et al. [18]	2018	95	74	82/13	94.8	Japan (MI)	51.6%	40	All	Pleural invasion, Foxp3 expression
Cattoni et al. [19]	2019	72	65	43/29	96	Italy and USA (MI)	57.6%	Na	I–IIIA	Tumour size > 3 cm
Roesel et al. [20]	2020	251	64	156/95	88.4	Germany (MI)	38.8	NA	All	Age, pN stage, chemotherapy
Shi et al. [21]	2020	106	65.6	105/5	51.4	China (SI)	36%	24.1	All	pT stage, NLR

*NA* not available, *SI* single institution, *MI* multiple institution

## Discussion

In 2004, based on cell morphology, the WHO classified pLCNEC, basaloid carcinoma, lymphoepithelioma-like carcinoma and clear-cell carcinoma as subtypes of large-cell lung cancer, but it also mentioned that pLCNEC had the characteristics of neuroendocrine carcinoma (NEC). In 2015, under improved recognition of pLCNEC and advancement of immunohistochemistry technology, the WHO classification system for lung cancer listed pLCNEC, typical carcinoid, atypical carcinoid and SCLC as four subtypes of pulmonary NECs [2, 22]. Currently, it is generally believed that among NECs, typical carcinoid and atypical carcinoid tumours have relatively good prognoses, while SCLC usually has a very poor prognosis. Due to its rarity and the uncertainty of its pathological classification in the past, pLCNEC is still not well understood. It was believed that its prognosis fell between that of carcinoid and SCLC and was more inclined towards that of SCLC, consistent with the classification of high-grade NECs along with SCLC [4, 23].

**Fig. 2:**
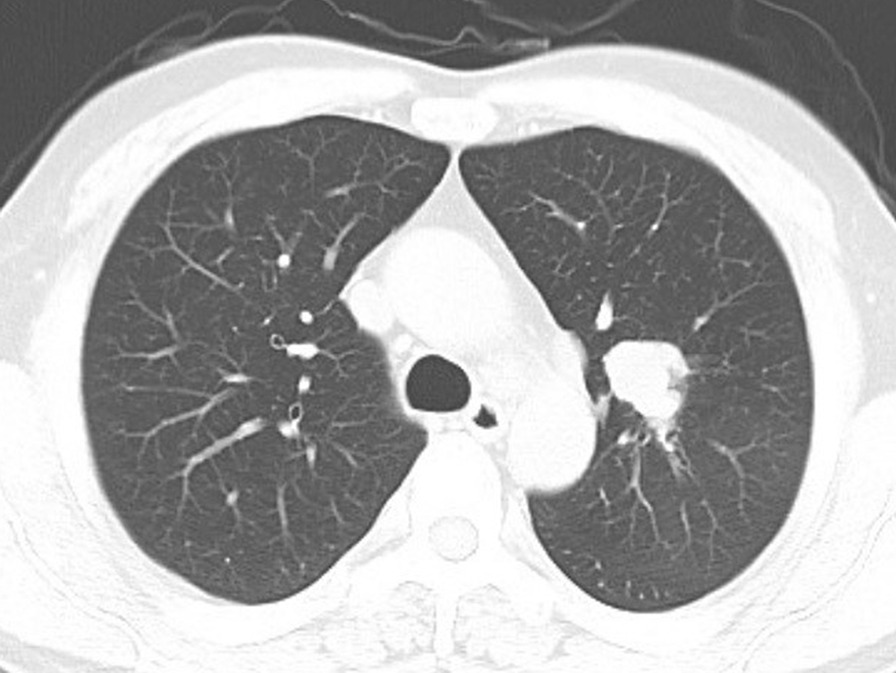
Imaging characteristics of peripheral pLCNEC. Chest CT showed a lobulated mass with a clear boundary and obvious short burrs

In this study, male patients accounted for 81.36%, 72.88% of the patients had a smoking history, the median age was 62 years (38–79 years) and peripheral lesions predominated (61.02%), in line with the basic characteristics reported previously [24–27] (Table 3). Due to the lack of clinical symptom specificity, imaging, especially chest CT, is an important means of detecting lesions. CT manifestations of peripheral lesions are lobulated nodules or masses with clear boundaries and short burrs, which are similar to those of peripheral SCLC and poorly differentiated adenocarcinoma [28, 29]. Figure 2 shows the peripheral pulmonary lesions (referring to lesions that occur in the distal end of the segmental bronchi) of one patient in this study, manifesting as a solid lobulated nodule with obvious short burrs. Nevertheless, no special imaging manifestation could be used to distinguish pLCNEC from other lung malignancies. PET/CT is usually used to assess the malignant tendency of the tumour and whether the patient has metastasis at other sites. However, independent studies on the use of PET/CT for LCNEC are lacking.

Preoperative diagnosis of pLCNEC was very difficult. Although the tumour markers CEA and ProGRP had certain diagnostic significance, their sensitivity and specificity were limited [24]. In this study, the common elevated tumour markers were CYFRA211 (18.8%), SCCAg (0.0%), NSE (34.4%), ProGRP (33.3%) and CEA (39.3%), but all lacked specificity. In addition, because fewer tissue specimens could be obtained by preoperative bronchoscopy or CT-guided biopsy, preoperative diagnosis of pLCNEC was relatively difficult [5, 30, 31]. Twenty patients enrolled in this study underwent preoperative bronchoscopy/CT-guided biopsy, but the biopsy pathologies of only three patients suggested pLCNEC. Thirty-one patients underwent an intraoperative frozen pathological examination, among which only one patient was diagnosed with pLCNEC. Since cell morphology, the mitotic phase and immunohistochemical markers are important indicators for identifying and classifying NECs, the diagnosis of pLCNEC can be confirmed only by careful interpretation of postoperative paraffin-embedded pathological sections. Consequently, the morphological identification and immunohistochemical interpretation of NECs in small biopsy specimens are very difficult [31, 32]. For this reason, the surgical indications for suspected pLCNEC should be appropriately widened. Even in the case of suspected metastasis, it is sometimes necessary to consider a surgical approach to obtain a sufficient amount of a tumour specimen to confirm the diagnosis and thus carry out targeted adjuvant therapy.

Surgery is currently an effective treatment for pLCNEC [5, 12, 33]. Although the subgroup analysis in this study suggested that there were no significant differences in OS between different surgeries (lobectomy, combined lobectomy and sublobar resection), a retrospective study [34] suggested that sublobar resection led to a relatively poor prognosis. In addition, the median tumour size of pLCNEC in this study was 3.0 cm; thus, anatomical lobectomy was recommended as the preferred surgical approach for pLCNEC.

For resectable pLCNEC, the reported prognosis varies significantly, with a 5-year survival rate ranging from 15 to 60%. Even for stage I patients, the 5-year survival rate varies significantly (18–88%). One possible reason for this variance is that pLCNEC is a rare disease, and the number of cases reported is relatively small. Misdiagnosis caused by a limited understanding of pLCNEC and a lack of immunohistochemical methods might also contribute to the varying survival rates [5, 31]. The median survival time of the patients in this study was 36 months (10.9–61.1 months), and the 5-year OS was 44.70%, which was consistent with the results of other surgery-focused studies (Table 3). It was reported that sex, treatments and tumour pathological stage were relevant factors affecting the survival of patients with pLCNEC [35, 36]. In this study, factors such as sex, age, lesion location, tumour stage and adjuvant therapy were analysed, and multivariate analysis showed that the N stage (HR 1.689, 95% CI 1.042 ~ 2.740, p = 0.034) and M stage (HR 6.712, 95% CI 2.229 ~ 20.211, p = 0.001) were independent prognostic factors. In addition, the OS rates of patients treated with different surgical approaches were significantly different (HR 0.407, 95% CI 0.195 ~ 0.851, p = 0.017), but a selection bias existed considering that minimally invasive surgical treatment was applied to only early-stage patient according to preoperative assessment. Although other scholars have performed prognostic analyses of factors such as the mitotic phase, Ki-67 index and immunohistochemical markers [5, 37], no definite conclusions have been drawn. As shown in Table 3, among the surgery-focused studies, the most frequently mentioned prognostic factors were still associated with the tumour stage or adjuvant therapy. The N and M stages were prognostic factors in this study, which also indicated that the tumour stage could preliminarily predict the treatment outcome and prognosis of pLCNEC.

Many studies have proposed new classifications of pLCNEC based on clinical, pathological and genotyping characteristics, which can be used to classify LCNEC into SCLC-like and NSCLC-like LCNEC [38–41]. This would be important for the prognostic evaluation and selection of adjuvant therapies for patients. For patients who could tolerate surgery, surgical treatment with or without chemotherapy prolonged their survival [36, 42, 43]. Regarding adjuvant chemotherapy, some studies suggested that compared with NSCLC chemotherapy regimens, SCLC chemotherapy regimens exerted a better therapeutic effect on pLCNEC [44]. However, another study showed that there were no significant differences in survival between the two regimens [45]. Therefore, different individualized treatment regimens based on different types should be considered to optimize patient survival [46]. Because previous studies on pLCNEC were single-centre, small and retrospective studies and corresponding guidelines were lacking, the adjuvant chemotherapies for patients in this study were not consistent. Most of the chemotherapy regimens were platinum-based combination therapies, including etoposide, vinorelbine, and docetaxel. Therefore, there was a bias in our evaluation of the effect of adjuvant therapy on prognosis, and the advantages of different chemotherapy regimens could not be evaluated. More robust and in-depth clinical evidence is needed for the treatment of pLCNEC.

Postoperative adjuvant radiotherapy mainly focuses on locally recurrent lesions, but patients with these lesions may have poor survival rates due to advanced-stage tumours. As shown in the survival analysis of this study, there were no significant differences in OS among patients who received different postoperative adjuvant therapies. Compared to patients who did not receive preoperative adjuvant therapy, those who did had worse OS (p = 0.037). Nevertheless, only five patients received preoperative chemotherapy, mainly due to advanced-stage tumours. In consideration of selection bias, we cannot conclude that preoperative chemotherapy can significantly affect survival. In addition, it remains controversial whether adjuvant therapy should be administered to stage I pLCNEC patients. The retrospective analysis in this study showed that for stage I pLCNEC patients, adjuvant chemotherapy did not provide an OS benefit. However, another study [47] suggested that for stage IB pLCNEC patients, adjuvant chemotherapy after complete resection of the tumour provided survival advantages, but patients in stage IA did not benefit from that. Another retrospective study [48] suggested that chemotherapy prolonged the survival of pLCNEC patients regardless of whether they were in stage IA or stage IB. Given the invasiveness of pLCNEC, we recommend that stage I patients receive adjuvant chemotherapy after surgery.

## Conclusions

In summary, pLCNEC is a rare and invasive malignancy with a poor prognosis. Diagnosis by puncture biopsy or frozen pathology was difficult, and a definite diagnosis relied on postoperative paraffin-embedded pathological sections with immunohistochemical staining. For resectable lesions, the combination of surgery and adjuvant therapy could have better therapeutic effects. The N and M stages were independent risk factors for prognosis. Due to the rarity of the disease, guidelines on diagnosis and treatment based on clinical trials are still lacking. Larger sample sizes and multicentric data should be utilized to draw more convincing conclusions.

## Methods

### Patients

From April 2004 to April 2019, we evaluated a consecutive series of patients who were diagnosed and treated at Peking Union Medical College Hospital. They were determined to have resectable lesions before surgery and confirmed to have pLCNEC by postoperative paraffin pathology. The clinical symptoms, imaging data, operation and comprehensive treatment patterns, pathological results and prognoses of all patients were analysed. All patients were managed by a multidisciplinary team consisting of senior thoracic surgeons, oncologists, pathologists, radiologists and radiotherapists at our hospital.

### Treatment method

All patients underwent surgery-based comprehensive treatment. According to the size and location of the tumour, lobectomy or sublobar resection plus lymphadenectomy was performed under thoracotomy or thoracoscopy. All patients were classified according to the AJCC/UICC 7th Edition TNM staging system.

We defined the perioperative period as the duration between the time of operation and one month after the operation.

### Statistical analysis

SPSS 22.0 software was used for data analysis. Normally distributed measurement data are expressed as the mean ± standard deviation, and nonnormally distributed data are expressed as the median (interquartile range). The Kaplan–Meier method and log-rank test were used to analyse the survival rates and prognostic factors. OS was calculated from the operation date to the patient's death or the last follow-up date. The variables with p < 0.05 in univariate analysis were included in a Cox regression model for multivariate analysis. Differences were deemed statistically significant when p < 0.05.

### Literature review

We reviewed the data of pLCNEC cohorts treated mainly by surgery, published and indexed on PubMed since 2004. Studies that focused mainly on nonsurgical treatments or patients with stage I pLCNEC were excluded. We summarized the treatment outcomes and related prognostic factors.

## Data Availability

All data generated or analysed during the study are included in this published article.

## Abbreviations

AT: Adjuvant therapy
Ct: Chemotherapy
CT: Computed tomography
CI: Confidence interval
HR: Hazard ratio
nd: Not done
NEC: Neuroendocrine carcinoma
NSCLC: Non-small-cell lung cancer
OS: Overall survival
pLCNEC: Pulmonary large-cell neuroendocrine carcinoma
PLT: Posterolateral thoracotomy
PUMCH: Peking Union Medical College Hospital
Rt: Radiotherapy
SCLC: Small-cell lung cancer
WHO: World Health Organization
VATS: Video-assisted thoracic surgery
